# Topological Cluster Analysis Reveals the Systemic Organization of the *Caenorhabditis elegans* Connectome

**DOI:** 10.1371/journal.pcbi.1001139

**Published:** 2011-05-19

**Authors:** Yunkyu Sohn, Myung-Kyu Choi, Yong-Yeol Ahn, Junho Lee, Jaeseung Jeong

**Affiliations:** 1 Department of Bio and Brain Engineering, Korea Advanced Institute of Science and Technology (KAIST), Daejeon, Republic of Korea; 2 Department of Political Science, University of California, San Diego, California, United States of America; 3 Research Center for Cellulomics, Institute of Molecular Biology and Genetics School of Biological Sciences, Department of Biophysics and Chemical Biology, Seoul National University, Seoul, Republic of Korea; 4 Center for Complex Network Research, Department of Physics, Northeastern University, Boston, Massachusetts, United States of America; 5 Center for Cancer Systems Biology, Dana-Farber Cancer Institute, Harvard University, Boston, Massachusetts, United States of America; University College London, United Kingdom

## Abstract

The modular organization of networks of individual neurons interwoven through synapses has not been fully explored due to the incredible complexity of the connectivity architecture. Here we use the modularity-based community detection method for directed, weighted networks to examine hierarchically organized modules in the complete wiring diagram (connectome) of *Caenorhabditis elegans* (*C. elegans*) and to investigate their topological properties. Incorporating bilateral symmetry of the network as an important cue for proper cluster assignment, we identified anatomical clusters in the *C. elegans* connectome, including a body-spanning cluster, which correspond to experimentally identified functional circuits. Moreover, the hierarchical organization of the five clusters explains the systemic cooperation (e.g., mechanosensation, chemosensation, and navigation) that occurs among the structurally segregated biological circuits to produce higher-order complex behaviors.

## Author Summary

*Caenorhabditis elegans* (*C. elegans*) is a tiny worm whose neuronal network is fully revealed. Since the modular organization in a network of individual neurons interwoven through synapses is not yet fully explored owing to incredibly complex connectivity architecture, this study is designed to investigate hierarchically organized modules in this complete wiring diagram (connectome) of this worm. We used the modularity-based community detection algorithm and found that *C. elegans* had 5 anatomical clusters in the *C. elegans* connectome, which corresponded to experimentally-identified functional circuits. We found that the hierarchical organization of the 5 clusters explains the systemic cooperation including mechanosensation, chemosensation, and navigation that occurs among the structurally-segregated biological circuits to produce higher-order complex behaviors.

## Introduction

The brain consists of a remarkably complex hierarchical structure ranging from ion channels of individual neurons to systemic neuronal networks of subsystems responsible for specific functions. To perform natural computation efficiently, the brain has evolved to have specialized modules with locally dense connections to integrate functions and produce complex behaviors. Because brain structure is closely related to function, an understanding of the topological structure of neuronal organization in the brain is crucial for insight into how neuronal networks perform their precise functions [1], [2], [3], . To uncover the neurobiological mechanisms of brain functions, mapping of the complete wiring diagram of a neural system has been attempted; this field is called connectomics [2], [9]. Although connectomics is presently at an early stage and data mining related to its application has only recently begun, the connectomics approach may eventually shed light on the fundamental principles underlying brain functions and the pathological mechanisms of neuropsychiatric disorders that arise from faulty wiring, such as schizophrenia and autism [2], [5], [6], [9], [10], [11], [12].

As accurate large-scale data describing the topology of networks become available in various fields, complex network analysis tools have been developed and applied. The study of complex networks involves the investigation of important topological features of a network with connections among its nodes that are neither purely regular nor purely random. This technique has been applied to complex networks of the real world, such as the worldwide web [13], metabolic networks [13], food webs [13], and neural [2], [5], [7] and social networks [2], [5], [7], [13], [14]. These complex networks have shown universal structural features including small-world properties [13], [14], power-law degree distributions [13], the existence of repeated local motifs [2], [15], and robustness and fragility against attacks [13]. Recently, the brain, a typical example of a complex network, was found to exhibit small-world topology from the microscopic level (e.g., the neuronal network of *C. elegans*) [14], [16] to the macroscopic level [2], [16], [17], [18]. Scale-free degree distributions are observed in fMRI-based voxel networks of human brains [2], and structural and functional motifs can be detected in the large-scale cortical networks of macaque monkeys and cats [2]. Robustness and fragility of brain structural networks with respect to lesions and diseases have also been examined quantitatively [7], [12], [18], [19].

Another significant issue in complex network analysis is the determination and characterization of the hierarchical cluster structure in a network, i.e., the appearance of densely connected groups of nodes with sparser connections among groups and their association at higher levels [20], [21], [22]. Topological clusters in brain structure may correspond to sets of distinct anatomical modules of neurons [2], [5], [6], [7], [23], [24], [25]. Detection of cluster structure in the brain is of critical importance because it provides valuable clues regarding the relationship between anatomical clusters and functional circuits. Such a relationship is based on the modular view of network dynamics, which assumes that different groups of neurons perform different functions with some degree of independence. Several studies have investigated the large-scale network structure of the mammalian cortex and its association with cortical function. Both the structure as a whole [2], [6], [7], [23], [25] and subsystems [24] of the brain have several distinct anatomical substrates (segregation) as well as functional connectivity (integration), implying an intimate association between structural clusters and functional modules at the macroscopic level [8], [17], [18]. However, because of the complexity of the connectivity architecture at the level of individual neurons, no studies have reported whether the connectome of an entire nervous system exhibits a hierarchical cluster structure.

Therefore, the aim of this study was to investigate the possible existence of cluster structure in the neuronal network of the entire nervous system of the nematode *Caenorhabditis elegans* (*C. elegans*) using the updated version of its wiring diagram (connectome) based on synaptic connection topology. The microscopic worm *C. elegans* has 302 neurons with approximately 8,000 synapses and is the only model organism in which the wiring diagram of the entire nervous system is almost completely known [3], [26]. We utilized this connectome to determine whether a network of individual neurons exhibits hierarchical cluster structure with non-uniform synaptic connections or a random network structure with homogeneous synaptic connections.

To detect a possible hierarchical cluster structure in the *C. elegans* connectome, we used the modularity-based community detection algorithm for directed weighted networks [20], [27]. Modularity is a quantitative measure defined as the number of edges falling within groups minus the expected number in an equivalent network with edges placed at random; positive values demonstrate the possible presence of cluster structure [20], [22], [27]. A significant advantage of the modularity-based community detection algorithm is that it can show a network to be indivisible (i.e., that it contains no cluster structure) if no true division of the network results in a positive modularity. Because a biological neural network is inherently directed and weighted, we implemented a recently introduced version of modularity function for directed and weighted networks and applied it to the directed weighted *C. elegans* connectome [27].

Although the modularity maximization approach of community detection has become the most popular and powerful method in the discipline, several recent studies have addressed some problems with this method [28], [29]. Because modularity optimization is known as an NP-complete problem, researchers have used a set of approximation heuristics to obtain a near-optimal community assignment vector without knowing the overall properties of the modularity landscape. However, Good et al. [29] examined the presence of an extremely rugged structure around the top of the modularity landscape through extensive computational validation of modular properties in many popular networks. This finding implies that the modularity maximization method may provide a great number of near-optimal vectors with very inhomogeneous characteristics and may not permit the determination of the goodness of each community vector without prior non-topological knowledge about node characteristics [28], [29].

In the case of the *C. elegans* connectome, however, we have a valid cue to overcome this issue: the information given by the bilateral functional symmetry of the neuronal cells as a constraint for optimization. Thus, we first show that the conventional implementation of modularity maximization using the spectral method and another popular greedy algorithm cannot produce biologically valid community assignment vectors. Second, we propose a novel scheme for constrained modularity optimization using a simulated annealing procedure. As a stochastic optimization method, this procedure allows a comparison of a diverse set of community assignment vectors for identification of a near-optimal partition. Through the extensive computational task of producing various community assignment vectors, we finally achieved a stable vector with the highest modularity value under given biological constraints. After detecting topological clusters in the *C. elegans* connectome, we investigated their network properties including spatial distribution of the neurons within clusters and their association with experimentally identified functional circuits.

## Materials and Methods

### Materials

We analyzed the one-dimensional spatial representation of the *C. elegans* wiring diagram recently published by Chen et al. [30] and Varshney et al. [31], which was updated from the dataset of White et al. [3] where connections were identified by electron microscopic reconstructions. The data contained information on the direction and number of connections via chemical synapses and electrical junctions among neurons in the entire nervous system as well as one-dimensional spatial positions of neurons (i.e., somal centers) along the anterior-posterior body axis. All connections between non-pharyngeal neurons were included except those of CANL/R and VC6, which did not have obvious synapses. Consequently, the model connectome had 279 neurons (pharyngeal and unconnected neurons excluded) with 6,393 chemical synapses and 890 electrical junctions. Data sets are available at http://www.wormatlas.org/neuronalwiring.html.

### Modularity-based community detection with external constraints using a simulated annealing method

In this study, the complete neuronal wiring diagram of *C. elegans* through chemical synapses and electrical junctions (connectome) was considered as a directed weighted network with basic topological attributes including degree, weight, and strength [32]. The degree equals the number of synaptic partner neurons of a neuron and the weight is the appropriate sum of synapses between specific neuronal partners. The strength represents the total weights of synaptic connections afferent to or efferent from a neuron. A weighted asymmetric adjacency matrix was devised to illustrate the synaptic connections between 279 neurons. The matrix size was accordingly 279×279 and the sum of the weights of each element represented the number of synapses from one neuron to another. The summed weight of all elements in the adjacency matrix (the total number of chemical synapses + double the total number of electrical junctions) was 8171.

To identify possible cluster structures in the *C. elegans* connectome, we used the modularity-based community detection algorithm for a directed and weighted network. The modularity value, *Q*, indicates the degree to which a given partition succeeds in maximizing intra-cluster weights and minimizing inter-cluster weights compared to a null model given a strength sequence. To detect clusters in a directed and weighted network, we implemented a directed network version of modularity, which is defined as follows:
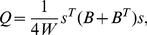
(1)

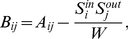
(2)where *A* is the adjacency matrix of a directed weighted network, *S_i_^in^* and *S_i_^out^* indicate incoming and outgoing strengths, respectively, of neuron *i* and 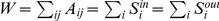 is the global sum of the weights of all dyads. Hence, *B_ij_* becomes a measure of the extent to which the number of connections from neuron *j* to neuron *i* are prominent in comparison with a randomized network.

After achieving the modularity function, we needed to search for a community assignment vector *s* that approximates the global maximal value of Q. To prevent the generation of suboptimal outcomes when using several deterministic algorithms, we implemented a stochastic hill climbing approach [21] to validate diverse near-optimal values. The algorithm is designed following the standard scheme of a metropolis algorithm, setting the objective function as a modularity function. First, we randomly assigned groups of nodes and flipped each nodal membership depending on computational temperature, *T,* and the marginal modularity gained by this action. As this optimization procedure repeats, *T* decreases so that we can search more limited areas with higher modularity values. After achieving an optimized vector with this individual nodal level manipulation, we repeated the same metropolis procedure in the level of communities. That is, merging two clusters with respect to the modularity gain. This method is the most accurate to date and contains assignment vectors in its pool of solutions that can be achieved by other community detection methods [21], [28].

In addition to this standard procedure, we considered an optimization method with external constraints. Using the information given by non-topological prior knowledge, we constrained the type of solutions [28], [29]. In the present study, the given constraint is the bilateral symmetry of neurons, which indicates that each bilateral pair should be classified in the same cluster. Thus, we searched for community assignment vectors within the global modularity landscape that satisfied this condition. This additional term can be easily implemented in the algorithm by providing a simultaneous cluster membership change constraint for each bilateral pair.

### Computing cluster proximity

We performed an additional analysis on the proximity of the obtained clusters. Following the second phase optimization procedure introduced in the fast unfolding algorithm [29], we built a new network whose nodes consisted of communities found by the initial simulated annealing algorithm and the link weights between the newly assigned nodes (i.e., summed values between inter-cluster weights). We then applied the same modularity maximization approach as described previously. This procedure revealed clusters of clusters where significant levels of clustering were present between previously obtained clusters.

## Results

### Identification of hierarchical clusters in the *C. elegans* connectome

To examine the presence of hierarchical cluster structure in the *C. elegans* connectome, we first estimated the modularity of this connectome within a framework of modularity-based community detection [20], [27]. This method seeks optimal divisions of the network into densely connected subgroups by maximizing the modularity *Q*. Because the *C. elegans* connectome had a power-law distribution of synaptic weights (Figure S1) and synaptic directions between neuronal connections, it was necessary to include the directionality and the number of synapses among neurons in the asymmetrically weighted elements of the adjacency matrix.

Although the modularity maximization approach of community detection has become a standard methodological means to detect possible community structures of networks, recent theoretical works have shown extreme degeneracy of solutions that produce near-optimal modularity values. One way to overcome this problem is to reduce the number of community assignment vectors using information given by prior knowledge of the node properties. Given that most bilateral neuronal pairs of *C. elegans* have similar functional roles [3], [26], [33] and accepting the principle of structure-function association in evolutionary biology [8], [29], structural clusters driven by an appropriate community detection method should not assign each member of a bilateral neuronal pair to a different structural cluster. We thus proposed a novel scheme to obtain an optimal community assignment vector. The simulated annealing method with external constraints in this study was utilized to find an optimal community assignment vector among the pool of solutions satisfying the bilateral symmetry condition.

**Figure 1:**
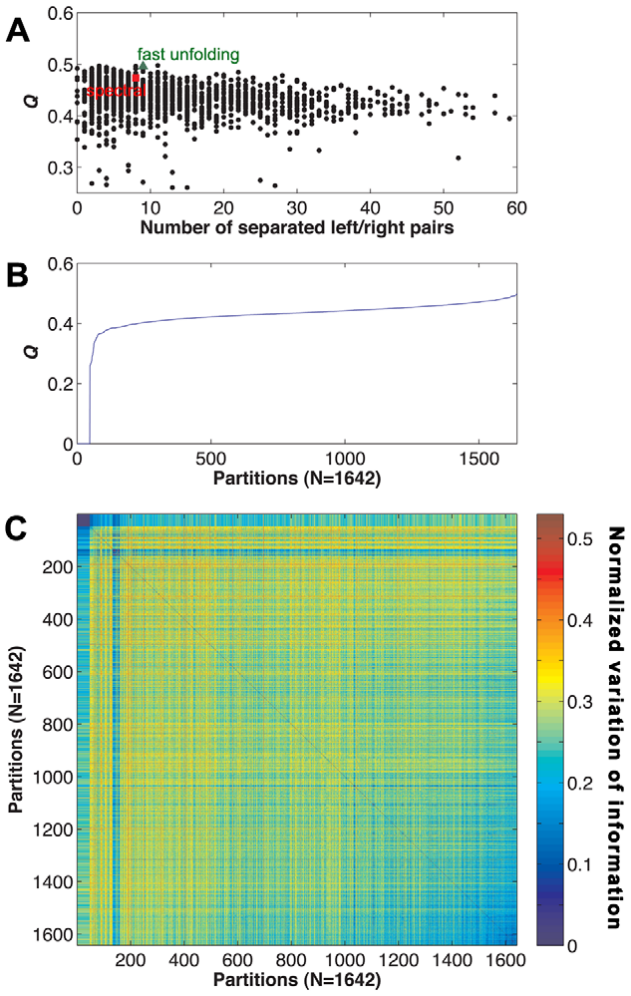
Diverse set of solutions obtained by the simulated annealing method. (*A*) Modularity and the number of separated bilateral pairs computed from various community assignment vectors obtained through the simulated annealing method without external constraints. The green triangle indicates the corresponding values for an assignment vector obtained using the fast unfolding method and the red square indicates the corresponding values for an assignment vector obtained using the spectral method. (*B*) Modularity value reordered for the various assignment vectors obtained through 1,642 trials of simulated annealing with external constraints. (*C*) Cluster similarity between the corresponding 1,642 vectors (reordered) measured by variation of information.

Figure 1A depicts the properties of diverse sets of solutions derived using the simulated annealing method without external constraints, the spectral detection method, and the fast unfolding algorithm. The spectral detection algorithm is one of the most popular algorithms because of its short computational time [22]. The fast unfolding algorithm is one of the most accurate and fast deterministic algorithms, resulting in a high modularity value [12], [28]. However, Figure 1A demonstrates that the set of solutions driven by the simulated annealing method [21] produced a higher modularity value than the other two solutions. Moreover, the solutions of the two methods had low biological plausibility. The number of separated left/right pairs of each community assignment vector was 8 and 9 of the 93 bilateral neuronal pairs (totally 186 neurons), respectively, whereas the simulated annealing algorithm produced solutions with less-separated bilateral pairs and comparable modularity values. Figure 1B and Figure 1C present the modularity values and the similarity of solutions derived using the simulated annealing method with external constraints. To show the stability of solutions in the high modularity region of the community assignment vectors with no separated bilateral pairs, we implemented a parameter called ‘variation of information’ that quantified the difference between two community assignment vectors. Variation of information between two partitions C and C*'* is defined as follows:
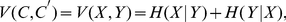
(3)where *X* and *Y* denote the vectors representing the cluster assignment of community divisions *C* and *C'*, respectively, *H(X|Y)* is the conditional entropy indicating the amount of additional information needed to describe *C* given *C'*, and *H(Y|X)* indicates the opposite condition. Consequently, *V(C,C’) = 0* indicates that two partitions are exactly identical and thus do not require any additional information to describe each other whereas a higher value indicates a greater difference in community assignment [34]. Because the maximum possible value of the difference between two partitions of a network having 279 nodes in terms of *V* is *log 279*, we rescaled the values to range from 0 to 1 by dividing the original value by *log 279* [*34*] *.* Figure 1C shows that the solutions obtained using the external constraint condition exhibited stable properties in the highest modularity region (*Q*>0.480) where each partition pair exhibited very low *V* values (0.122±0.002).

Through an extensive computational analysis (over 10,000 trials of simulated annealing with external constraints), we obtained an optimal cluster assignment with *Q* = 0.490, resulting in no separated bilateral neuronal pairs. This value was substantially higher than the average *Q* (0.283±0.009) of null networks obtained by swapping synaptic connections between neuronal pairs of the original network while preserving the out-strengths of the neurons [35]. With this maximal *Q* value, we found 5 distinct anatomical clusters in the *C. elegans* connectome. This result indicates that, among the possible connection distributions in the original strength sequence, the neuronal architecture of *C. elegans* exhibits a statistically significant modular structure.

We also measured the topological proximity between the obtained clusters to determine whether a hierarchical relationship was present between them. Following the second phase optimization procedure of the fast unfolding algorithm, we built a new network whose nodes are communities found by the initial simulated annealing algorithm. ‘Link weights’ between the newly assigned nodes consist of summed values between inter-cluster weights. By applying the modularity maximization algorithm to this new network, we showed that the previously obtained 5 clusters further clustered into 2 clusters in the higher level. This procedure allowed us to obtain a hierarchical dendrogram of the 5 modular clusters. Former branching was assigned a nomenclature of 1 (2 in the left digit), and later branching was called 1 (or 2 rightward). For instance, cluster 11, 12 and 13 have the same mother. Out of 279 neurons, 57 neurons were in cluster 11, 79 in cluster 12, 14 in cluster 13, 74 in cluster 21, and 55 in cluster 22. Cluster information for each neuron is listed in the Table S1.

**Figure 2:**
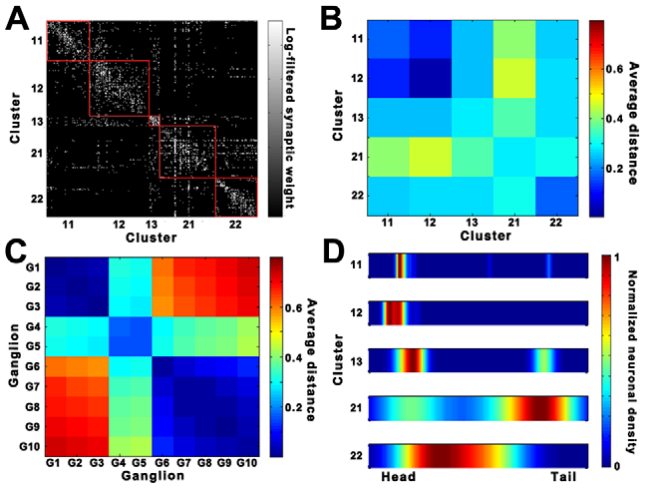
Optimal divisions of the *C. elegans* connectome using the modularity-based community detection algorithm. (*A*) Reordered adjacency matrix with cluster borders. The synaptic weights are log-filtered. Cluster boundaries are colored in red. (*B*) Inter-cluster distance graph. Neurons are grouped by the cluster that they belong to. The average distance between all pairs of a neuron in cluster *i* and a neuron in cluster *j* is calculated (between every two neurons of the same cluster for a diagonal element). (*C*) Inter-ganglion distance graph computed using the procedure of (*B*) based on ganglia. G1: anterior ganglion, G2: dorsal ganglion, G3: lateral ganglion, G4: ventral ganglion, G5: retrovesicular ganglion, 6: posterolateral ganglion, G7: ventral cord neuron group, G8: pre-anal ganglion, G9: dorsorectal ganglion, G10: lumbar ganglion. (*D*) Spatial density distributions for clusters along the anterior-posterior body axis.

The topological relationships based on synaptic connections within and among the clusters are demonstrated in the reordered adjacency matrix of the *C. elegans* connectome in Figure 2*A*. Although the off-diagonal elements of the adjacency matrix for inter-cluster links had low values, large values of the diagonal elements in Figure 2*A* indicate that most of the links were intra-cluster for each of 5 clusters. Figure 2*A* also provides information on the hierarchical relationship between the clusters. As illustrated, we observed many ties across the clusters that depended on hierarchical proximity: cluster 11, 12, and 13 formed a grand cluster and cluster 21 and 22 formed another grand cluster. The complete hierarchical dendrogram of the entire neurons, which accords with this cluster level hierarchical relationship, is presented in the supplementary information (Figure S6).

The fact that the length of *C. elegans* is about ten times greater than its diameter allowed us to consider the positional distributions of neurons within each cluster in one dimension [3], [26], [30], [33]. Figure 2*B* shows the average distances between the somata of neurons within each cluster and between clusters. Between inter-cluster neurons, the average distance was smaller than 0.5 unit length (Figure 2B), whereas the two largest proximal ganglia groups (groups of neurons aggregated based on the positions of their cell bodies), G1 to G3 and G6 to G10, were located at large average distances from each other (Figure 2C). While *C. elegans* neurons are spatially concentrated in a manner related to their ganglionic affiliation, we failed to observe a strong spatial localization of neurons belonging to the same cluster, except for those in clusters 11 and 12. We estimated the density of the somata of all neurons on the horizontal plane along the anterior-posterior body axis of the animal (Figure 2D). We found that clusters 11 and 12 were densely localized in the head. In contrast to the extreme spatial localization of ganglia (Figure 2D) [36], we detected a body-spanning cluster, cluster 22, that was distributed from the head to the tail of the worm's body (Figure 2D). We also noted the presence of clusters 13 and 21, which loosely spanned the anterior and posterior parts of the body, respectively.

### Membership properties of structural clusters

**Figure 3:**
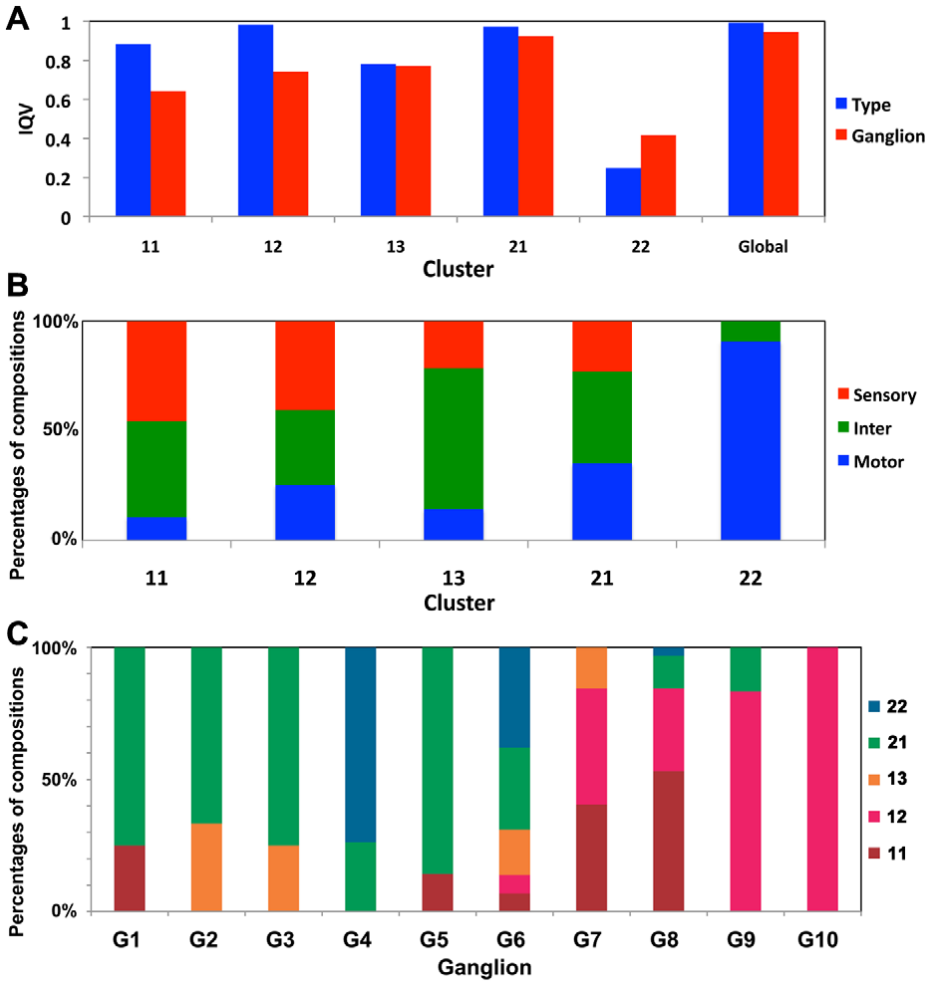
Neuronal composition of the structural clusters. (*A*) The IQV scores of all clusters with respect to the neuronal type and ganglion composition. (*B*) Compositions of neuronal types for each cluster. ‘Sensory,’ ‘Inter,’ and ‘Motor’ denote sensory neurons, interneurons, and motor neurons, respectively. (*C*) Cluster membership compositions of ganglia.

We examined the compositions of neuronal types and ganglionic affiliations of neurons within clusters as shown in Figure 3. The diversity of neuronal types for a cluster was quantitatively measured using the index of qualitative variation (IQV) (see SI for detailed information) [37]. The IQV measures the heterogeneity of composition in a cluster; high IQV scores for a cluster indicate that the cluster is composed of various neuronal types or ganglionic neurons. In other words, if a set is composed of only a few dominant types, the IQV approaches 0, and it reaches 1 in the opposite case. Except for cluster 22, the clusters exhibited IQV values ranging from 0.78 to 0.98, indicating that the majority of the clusters did not possess dominant neuronal types (Figure 3A). In addition, four of 5 clusters did not display dominant neurotransmitter types (Figure S2). The single exception was cluster 22, which consisted of 90% motor neurons and had an IQV value of 0.25 (Figure 3B) (also see Figure S4). All ganglia exhibited a rich diversity of cluster affiliations in their membership (Figure 3*A and C*), indicating that low levels of overlaps exist between ganglia and cluster assignments. Quantitatively, the IQV between ganglia and cluster assignments was 0.36, indicating a low level of correlation between the two assignments.

### Functional cartography of the *C. elegans* connectome

**Figure 4:**
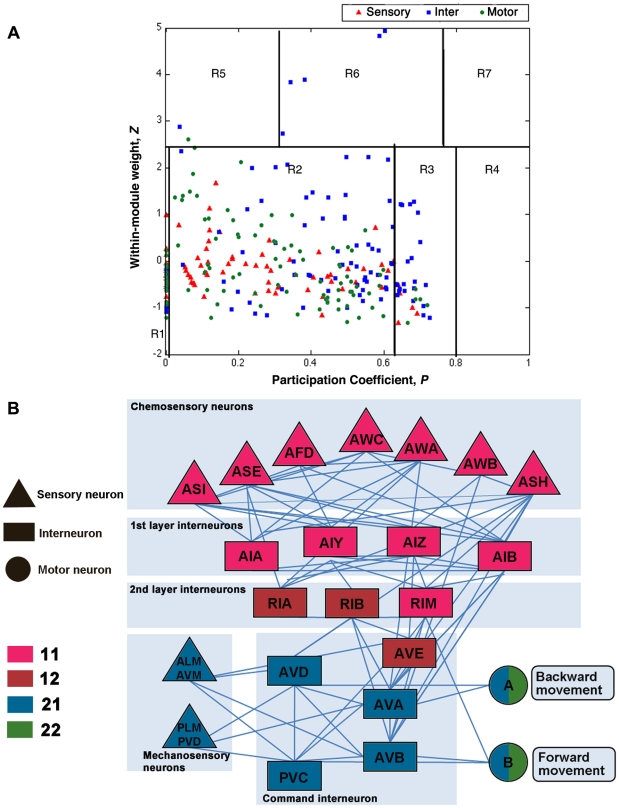
Functional implications of the derived clusters. (*A*) Functional cartography of neurons in the *C. elegans* connectome using the within-module weight (*Z*) and participation coefficient (*P*) of each neuron. The neurons within each region can be defined as: (R1) ultra-peripheral nodes; (R2) peripheral nodes; (R3) non-hub connector nodes; (R4) non-hub kinless nodes; (R5) provincial hubs; (R6) connector hubs; and (R7) kinless nodes, based on the conventional rules for classification. The exact value ranges of *P* and *Z* for each class are denoted in Text S1 (Table S2). (*B*) Cluster affiliation of neuronal pairs responsible for the behavior of a worm identified by previous biological experiments. The color of each neuronal pair indicates its affiliation to a specific cluster.

Classification of nodes using their intra- and inter-cluster connections has been used for the cartographic representation of complex networks [21]. To determine whether the characteristics of neurons in the context of a modular network are associated with their biological functions, we estimated the within-module weight (*Z*) and participation coefficient (*P*) of all neurons in the *C. elegans* connectome. The within-module weight (Z) evaluates how strongly a neuron is connected to other neurons within its cluster, and the participation coefficient (*P*) quantifies how extensively the connections of a neuron are distributed among different clusters. By plotting the *P* and *Z* values for each neuron in a two-dimensional plane, we characterized each neuron as either a provincial or peripheral node, a hub, or a node with few within-module degrees (see SI for detailed information). The *P* and *Z* values for each neuron are listed in the Table S3. According to the classification criteria suggested by Guimera and Amaral [21], we found that most of the neurons belonged to groups of ultra-peripheral nodes (role R1, 42 out of 279), peripheral nodes (role R2, 196 out of 279) or non-hub connector nodes (role R3, 34 out of 279) (Figure 4*A*). Neurons with the highest *P* values (*P*>0.62) were concentrated in the non-hub connector class (role R3) of low *Z* values (-2<*Z*<2) rather than in the connector hub class (Role R6). This result indicates that the clusters in the *C. elegans* connectome are connected via internal peripheral members. Interestingly, most neurons (86%) classified as ultra-peripheral nodes (role R1) with *P* = 0 were sensory or motor neurons, whereas all of the neurons classified as connector hubs (role R6) were command interneurons (AVA, AVB, PVC)[3]. These results suggest that interneurons play an important role both in connecting other neurons to form a cluster and in bridging between clusters.

### Association between topological clusters and functional circuits

To determine whether our topological clusters have functional relevance, we investigated how topological clusters were associated with functional neural circuits already studied experimentally. In Figure 4*B*, we present a diagram focusing on the two circuits having the largest memberships: mechanosensation and chemosensation [26], [38], [39].

*C. elegans* responds to various mechanical cues by means of specific sensory neurons. ALM, AVM, PLM, and PVD have roles in sensing mechanical touch [40], [41], [42]. These mechanosensory neurons belonged to cluster 21 (Figure 4*B*). Cluster 21 also contained some command interneurons, AVD and PVC, which are responsible for transmitting mechanosensory inputs to motor neurons [40], [41], [42] (Figure 4*B*).

In the case of chemosensation, chemical signals are sensed by different sets of neurons. For example, the neurons AWC and ASE have roles in sensing volatile and water-soluble compounds, respectively [43], [44]. AIA, AIY, AIZ, and AIB are the 1^st^ layer interneurons that receive synaptic inputs directly from sensory neurons; together with the chemosensory neurons, they belong to cluster 11. The 1^st^ layer interneurons direct their outputs onto the 2^nd^ layer interneurons (RIA, RIB, RIM, and SMB), which belong to clusters 11 and 12.

When chemical/mechanical signals are processed and transmitted within the *C. elegans* neural networks, the ultimate outcome is movement and behavior mediated by the motor neurons connected to body muscles. For instance, in chemosensation, signals processed in the 2^nd^ layer interneurons and mechanosensory neurons pass onto motor neurons via command interneurons (AVD and PVC) [3], [38], [39]. When body muscles contract, class A motor neurons are important for backward movement, while class B motor neurons have a role in forward movement [40], [41], [42] (also see Figure S5 and Table S4). All of the class A and B motor neurons belonged to cluster 22 (13 of 21 class A and 12 of 18 class B neurons) and cluster 21 (8 of 21 class A and 6 of 18 class B neurons). Interestingly, AVA neurons, the command interneurons that are important for backward movement [40], [41], [42], and AVB neurons, [40], [41], [42] responsible for forward movement, belonged to cluster 21 together with some class A and B motor neurons, indicating that the body-spanning clusters (21 and 22) are responsible for forward and backward movement. Taken together, these observations suggest that the topological clusters we observed are closely associated with functional circuits in the *C. elegans* connectome (Figure 4).

To quantitatively demonstrate the discriminative power of the current community assignment, we used a boot-strap sample t-test. The aim of this analysis was to determine whether a randomly assigned community vector with the same cluster size distribution would show a similar level of discriminative power for the circuits represented in Figure 4B as the optimized solution. By assigning the functional groups of neurons as chemosensory neurons, 1st layer interneurons, 2nd layer interneurons, mechanosensory neurons, command interneurons, and class A and B motor neurons, we measured the extent to which the original community assignment vector was consistent with the functional grouping of the 84 neurons. The resulting V value between the optimized assignment vector and the functional grouping was 0.348, whereas the mean value between randomized vectors with the same cluster size distribution and the functional grouping was 0.893 (±0.002). This result implies that the optimized vector's concordance with the functional groups was significant at the 99% confidence level.

### Systemic integration among clusters to produce more complex behaviors

**Figure 5:**
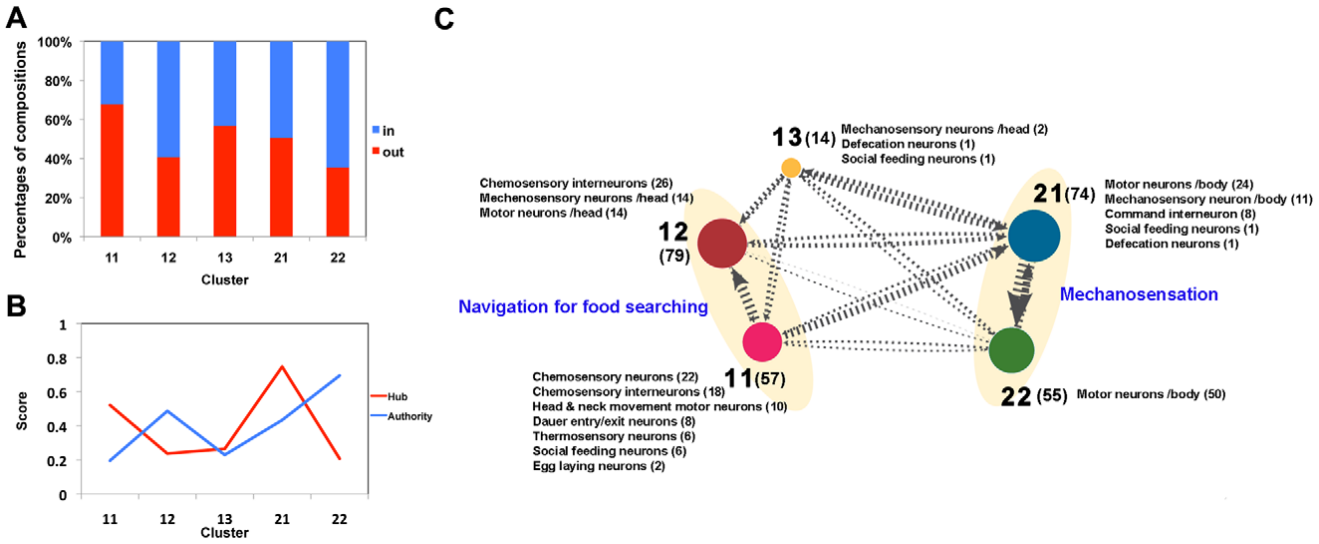
The structural relationship between 5 hierarchical clusters. (*A*) The ratio of in and out synapses for each cluster toward other clusters. (*B*) Hub and authority scores of each cluster. A cluster with a high hub score contains many outward synapses of high quality, whereas a cluster with a high authority score has high-quality inward synapses. (*C*) Representation of the hierarchical relationship between the clusters with their biological functions. The thickness of each edge and arrow is proportional to the synaptic weight between each dyad. The size of the circle representing a cluster is proportional to the intra-cluster synaptic weight of the cluster. The numbers in parentheses indicate numbers of neurons.

To examine whether the deduced information flow was reflected in the clusters at the level of synapse directionality, we estimated the inward/outward synapse ratio of each cluster toward other clusters. We considered that cluster 11, the major members of which are sensory neurons, was the information-producing cluster and thus should have mostly outward synapses. Indeed, 68% of cluster 11 neurons had outward synaptic weights (Figure 5A). On the contrary, cluster 22, which was the information-receiving cluster (i.e., composed of motor neurons), had mainly inward synapses (65% having inward synaptic weights). Clusters 12, 13 and 21, which possessed comparable numbers of neuronal types (clusters 12 and 21) or were predominantly composed of interneurons, exhibited balanced levels of inward and outward synaptic weights.

To investigate the information flow between clusters in terms of complex networks, we estimated ‘hub and authority scores’ of the clusters in the *C. elegans* connectome. Hub and authority scores measure the quality of the connections each node contains and show the significance of nodes in a directed network in a dynamic regime (see SI for detailed information) [45]. In our cluster-to-cluster network analysis, a cluster with a high hub score is linked through outward synapses to clusters having many inward synapses. Conversely, authoritative clusters have many inward synapses from clusters that bridge to them through outward synapses. We found that the authority scores of the clusters were proportional to the intensity of inward synaptic weights (Figure 5A and B). Thus, the body-spanning cluster 22, whose members are predominantly motor neurons, acted as an ‘authority’ receiving information from hub clusters to produce consequential behaviors. In contrast, the hub scores of the clusters were not strongly related to their outward synaptic weights. The cluster with the highest outward weight ratio (cluster 11) was not the most prestigious hub cluster, whereas the cluster with the highest hub score (cluster 21) had equivalent degrees of in and out synapses. This result may reflect the presence of indirect connections from the information-producing cluster 11 to the information-receiving parts of various clusters. In contrast, clusters 21 and 22, which exchange connections with each other, are motor neuronal clusters with direct synaptic connections (Figure 5C).

With respect to functional relevance, our topological analysis provided a hierarchical model of the information flow among structural clusters (Figure 5*C*). For example, the hierarchically close clusters 11 and 12 were functionally associated with each other for chemosensory behavior (navigation for food searching) [39]. As noted, cluster 11 contained mostly chemosensory neurons and 1^st^ layer interneurons, while cluster 12 contained 2^nd^ layer interneurons and motor neurons responsible for head and neck movement. This hierarchical relevance was also apparent between clusters 21 and 22 for the behavior of anterior touch response. Interestingly, our prediction of the information processing procedure between the clusters agreed with the nodal-level depiction of information processing hierarchy derived using a Laplacian matrix analysis [31]. We computed the cluster-level mean value of information processing hierarchy introduced by Varshney et al. [31]. This measure describes a chain of information producers and receivers in a one-dimensional axis using information obtained from complex recursive structural interactions between the neurons in the connectome. As a result, information producers have a high level of parametric value and receivers have a low level of parametric value. The average values of this parameter for neurons belonging to each cluster were as follows: cluster 11 (0.65±0.55) > cluster 13 (0.17±0.54) > cluster 12 (0.03±0.37) > cluster 21 (0.01±0.83)>22 (−0.77±0.70). This trend implies that the flow of information follows the path of cluster 11 → 13 → 12 → 21 →22. Using the same measure of information hierarchy, we found that motor neurons belonging to cluster 21 were located in an earlier processing phase of the information hierarchy than the motor neurons of cluster 22. The mean value of this parameter for motor neurons of cluster 21 was 0.15±0.66, whereas the mean value for the neurons belonging to cluster 22 was 0.23±0.67. The value of this parameter also tended to grow as the location of a motor neuron moved posteriorly (Figure S3), supporting our claim that posterior motor neurons are located at an earlier stage of information processing than anterior motor neurons. From the inward/outward synaptic ratios and the directionality of information flow between clusters, it is plausible to suggest that information flow among the structural clusters identified in this study occurs as follows: (1) chemosensation: 11 → 12 → head movement for changing direction, 11→ 12 → 21 → 22 → body movement; (2) mechanosensation: 21 → 22 → body movement. To summarize, the structural clusters indentified in this study appear to serve as a cohesive sub-module for information processing at various stages.

## Discussion

*C. elegans* is the only organism in which all synapses in the nervous system have been anatomically elucidated. Numerous studies have used this information to investigate how neuronal connections are related to their functions. However, few attempts have been made to identify structurally meaningful clusters by considering the complete wiring diagram of synaptic connections without any prior knowledge or other bias. Analysis of the *C. elegans* connectome revealed the existence of 5 topological clusters, including a body-spanning cluster, on the individual neuronal level, each of which corresponds to experimentally identified functional circuits. The hierarchical relationships between the five clusters define the systemic cooperation (e.g., mechanosensation, chemosensation, and navigation) between structurally segregated biological circuits toward higher-order complex behaviors. This study explicitly shows structural substrates of functional systems in a micro-scale connectome, which may provide experimentalists with possible predictions for functions of novel circuits in the *C. elegans* connectome.

What is the significance of the existence of distinct structural clusters in the *C. elegans* connectome? We show that the nervous system of the nematode, though seemingly simple, is organized into distinct functional modules. A ganglion contains neurons belonging to distinct clusters, suggesting that a ganglion is a simple collection of neurons with their somata lying near each other but also with different functional roles. Thus, synaptic connections make a greater contribution to the biological function of the *C. elegans* connectome than does the physical location of neuronal cell bodies.

We found that each cluster identified through topological clustering exhibited close relationships with its function in neural circuits, supporting our speculation that clustering analysis would be helpful in elucidating the functions of unidentified neurons. Supporting this idea, previous findings of neuronal ablation experiments are consistent with our clustering data. Most command interneurons, except for AVE, are included in cluster 21 (Figure 4*B*). Cluster 21 also contains the mechanosensory neurons ALM and PLM (Figure 4B). If ALM and PLM neurons are ablated, the worms do not respond to anterior and posterior body touch, respectively. Cluster analysis suggests that the command interneurons contained in cluster 21 are involved in mechanosensation. Consistent with this conclusion, when AVD or PVC neurons were ablated, the worms could not sense anterior or posterior body touch, respectively [40], [42]. Among the command interneurons, only AVE neurons belonged to cluster 12, which is consistent with the previous finding that ablation of the AVE pair alone did not result in any locomotion defect [42]. It is possible that, unlike other command interneurons, AVE neurons are involved in connecting chemosensory signals to motor circuits.

Using computational output, it is possible to make important predictions about the roles of neurons whose functions have not yet been examined or elucidated. For example, because sensory neurons in cluster 11 are experimentally known to be involved in chemosensation while sensory neurons in cluster 12 are involved in mechanosensation, we can hypothesize that unknown neurons, such as ADA neurons in cluster 11 and IL2 neurons in cluster 12, may be involved in chemosensation and mechanosensation, respectively. These hypotheses can be experimentally examined. The approach we employed in this study can be extended to other more complex organisms and represents a strong methodology for determining the functional properties of the connectome of other animals.

In addition, it will be feasible to test hypotheses based on our information flow using optogenetic methods and neural imaging [46]. For example, after expressing and activating channel rhodopsin proteins in motor neurons belonging to cluster 21, which are mostly located in the posterior region of the body, one could examine whether neural information can be transmitted to the neurons in cluster 22. This kind of approach may help to dissect the mechanism of locomotion in more detail. Ablation experiments could also be employed in addition to the inhibitory method using halorhodopsin.

Our findings must be interpreted in light of the limitations of this study. Because we lack knowledge on circuit-level information processing in *C. elegans* neuronal function at present, further validation based on biological experiments is necessary to confirm our findings and to build detailed computational methods for better predictions. Although we analyzed a recent version of the connectome as a directed weighted network, derivation of a more appropriate adjacency matrix of the connectome remains a goal for future theoretical studies. Because the *C. elegans* connectome contains distinct types of chemical synapses with excitatory or inhibitory synaptic effects, the development of a plausible framework for estimating the correct numbers for each element of the adjacency matrix will be required.

## Supporting Information

Figure S1 Weight distribution of the *C. elegans* connectome on the log-log scale with power-law fitting [2]. The scaling exponent of this distribution, α, is 2.72. This implies that the synaptic dyads in the network possess very uneven connection weights.
(0.06 MB TIF)

Figure S2 Neurotransmitter composition ratio for each cluster. The data were collected from the worm atlas website (http://www.wormatlas.org/neurons.htm/NTs.htm).
(0.28 MB TIF)

Figure S3 Correlation between anterior to posterior motor neuron index and the information hierarchy level parameter. The location of a motor neuron gets close to posterior as the number in the index of its label increases. We plotted the mean value of the parameter value for each neuron group having a same number index (ex. AS01, DA01, DV01, DD01, VA01, VB01, VC01 and VD01). The figure illustrates the presence of positive correlation between the two values (Pearson correlation  =  0.3865) having a slightly decreasing trend in the most anterior part of the worm.
(0.12 MB TIF)

Figure S4 Fraction of poly-synaptic weights/chemical synaptic weights minus the fraction in the overall network (0.55) represented in the cluster to cluster connection matrix.
(0.12 MB TIF)

Figure S5 Association between muscles and the clusters. (A) Strength of attachment of each muscle to the clusters represented by synaptic weight linked to the clusters. (B) Distribution of diversity of linked clusters for each neuron measured using IQV.
(0.15 MB TIF)

Figure S6 The complete community hierarchy of the 279 neurons.
(0.12 MB TIF)

Table S1 The complete list of neuronal affiliation for each cluster in the *C. elegans* connectome.
(0.03 MB XLS)

Table S2 Classification of the neurons in the *C. elegans* connectome by their topological roles.
(0.04 MB XLS)

Table S3 Values of within-module weights Z and participation coefficients P of each neuron in the *C. elegans* connectome with basic statistical analysis.
(0.04 MB XLS)

Table S4 Association between muscles and the clusters. The lists of IQV values and dominant cluster association information of each muscle.
(0.03 MB XLS)

Text S1 Supporting Information
(0.07 MB DOC)
