# Supplementary figures and images for: Topological Cluster Analysis Reveals the Systemic Organization of the Caenorhabditis elegans Connectome
Source: PLoS Comput Biol. 2011 May 19;7(5):e1001139. doi: 10.1371/journal.pcbi.1001139 (PMC3098222; doi:10.1371/journal.pcbi.1001139)

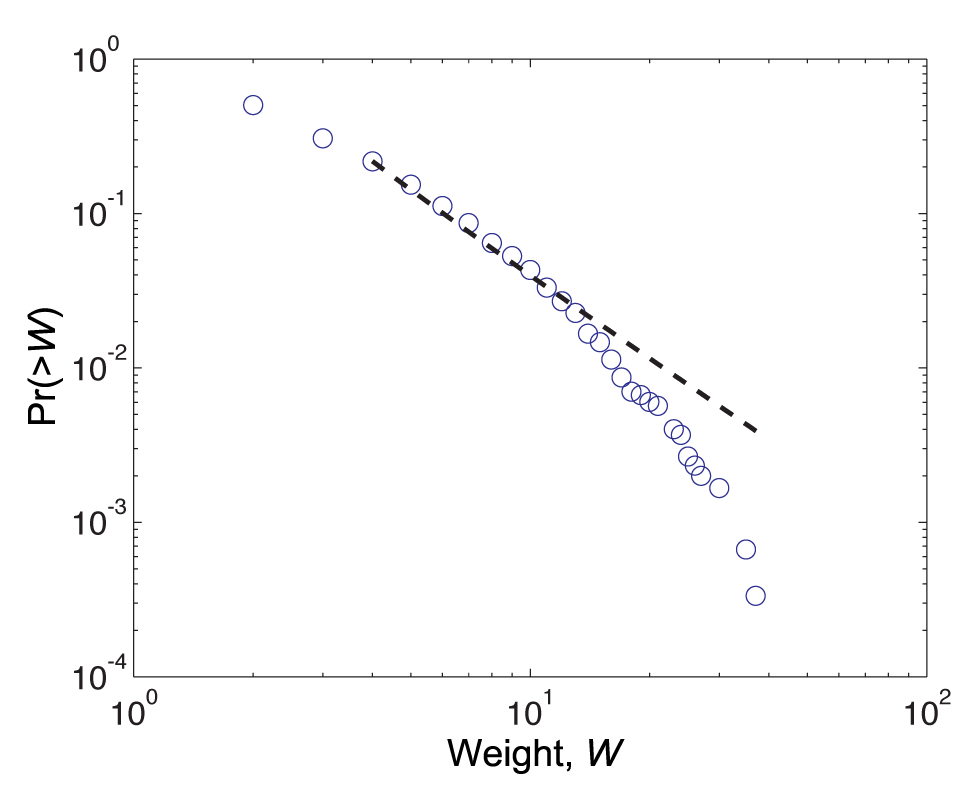

Supplement: Figure S1 — Weight distribution of the C. elegans connectome on the log-log scale with power-law fitting [2]. The scaling exponent of this distribution, α, is 2.72. This implies that the synaptic dyads in the network possess very uneven connection weights. (0.06 MB TIF) [file pcbi.1001139.s001.tif]

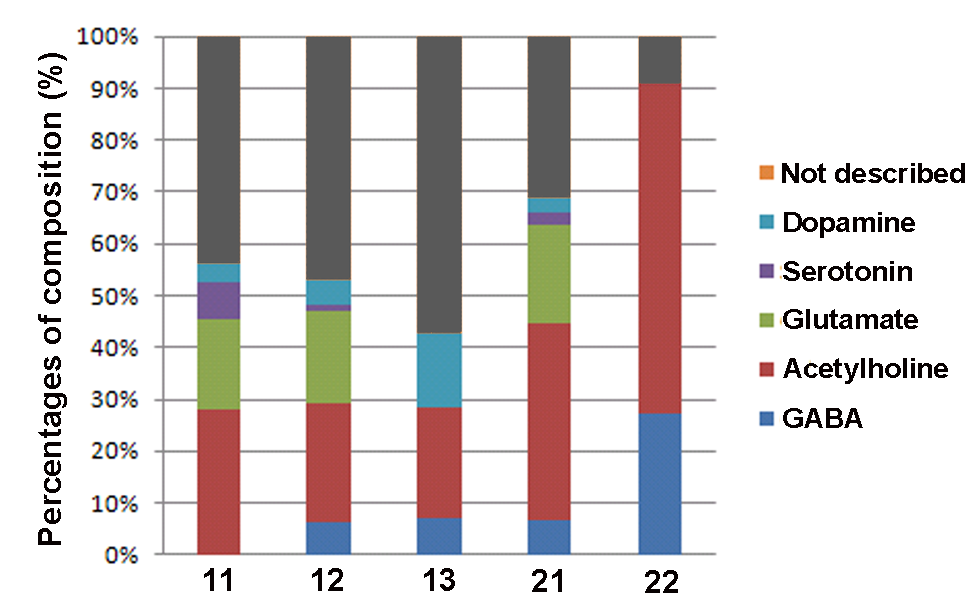

Supplement: Figure S2 — Neurotransmitter composition ratio for each cluster. The data were collected from the worm atlas website (http://www.wormatlas.org/neurons.htm/NTs.htm). (0.28 MB TIF) [file pcbi.1001139.s002.tif]

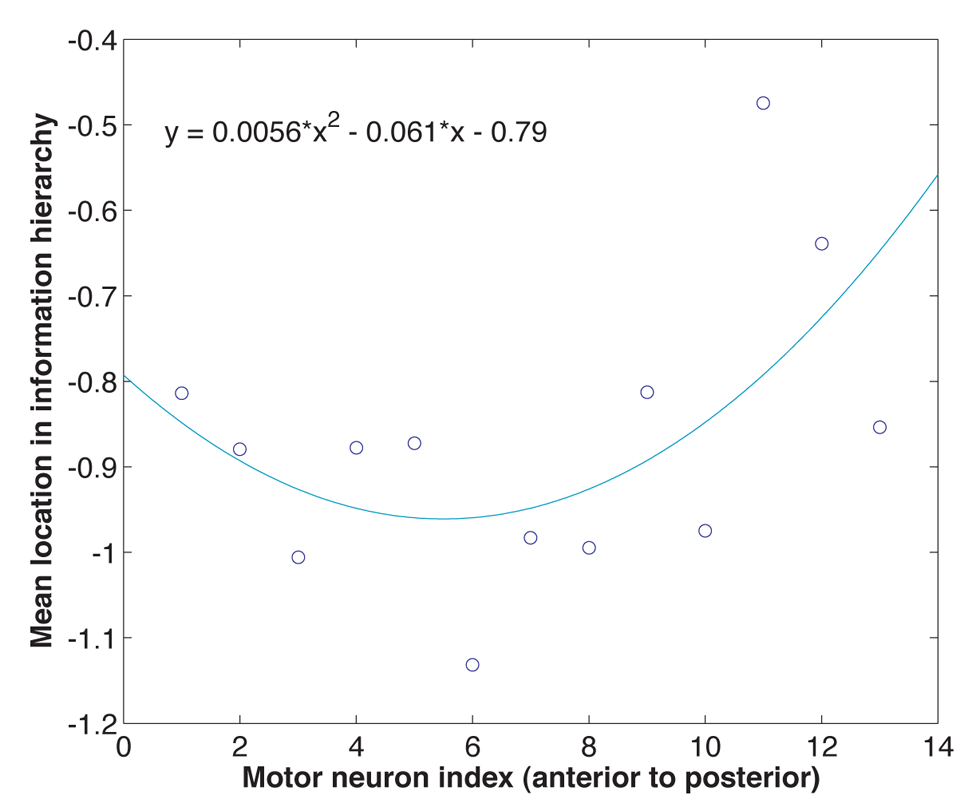

Supplement: Figure S3 — Correlation between anterior to posterior motor neuron index and the information hierarchy level parameter. The location of a motor neuron gets close to posterior as the number in the index of its label increases. We plotted the mean value of the parameter value for each neuron group having a same number index (ex. AS01, DA01, DV01, DD01, VA01, VB01, VC01 and VD01). The figure illustrates the presence of positive correlation between the two values (Pearson correlation = 0.3865) having a slightly decreasing trend in the most anterior part of the worm. (0.12 MB TIF) [file pcbi.1001139.s003.tif]

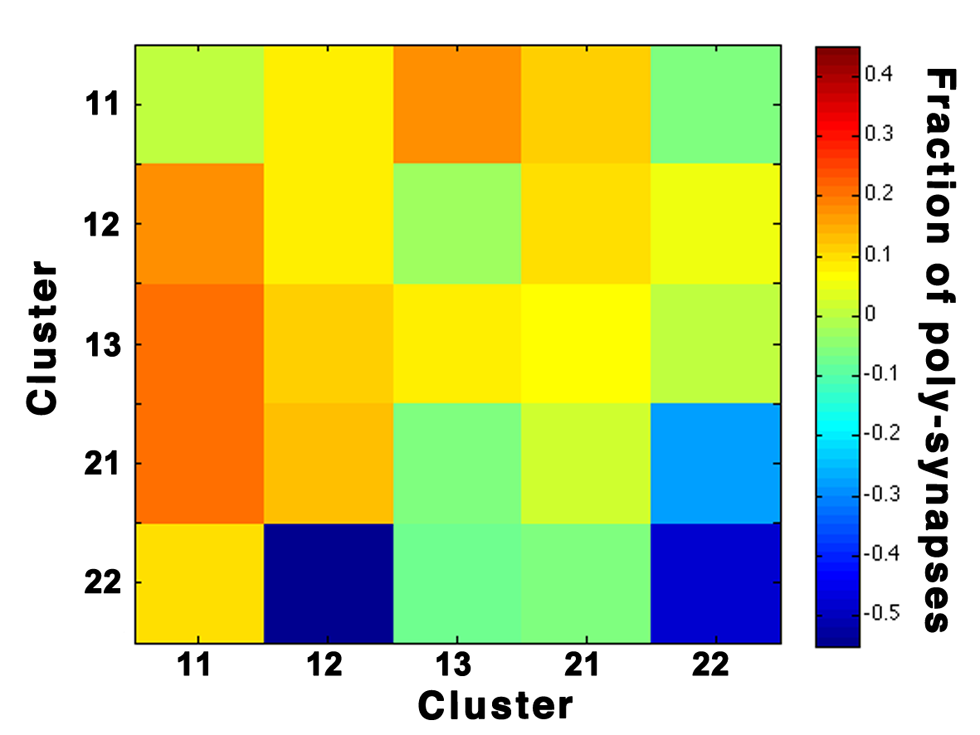

Supplement: Figure S4 — Fraction of poly-synaptic weights/chemical synaptic weights minus the fraction in the overall network (0.55) represented in the cluster to cluster connection matrix. (0.12 MB TIF) [file pcbi.1001139.s004.tif]

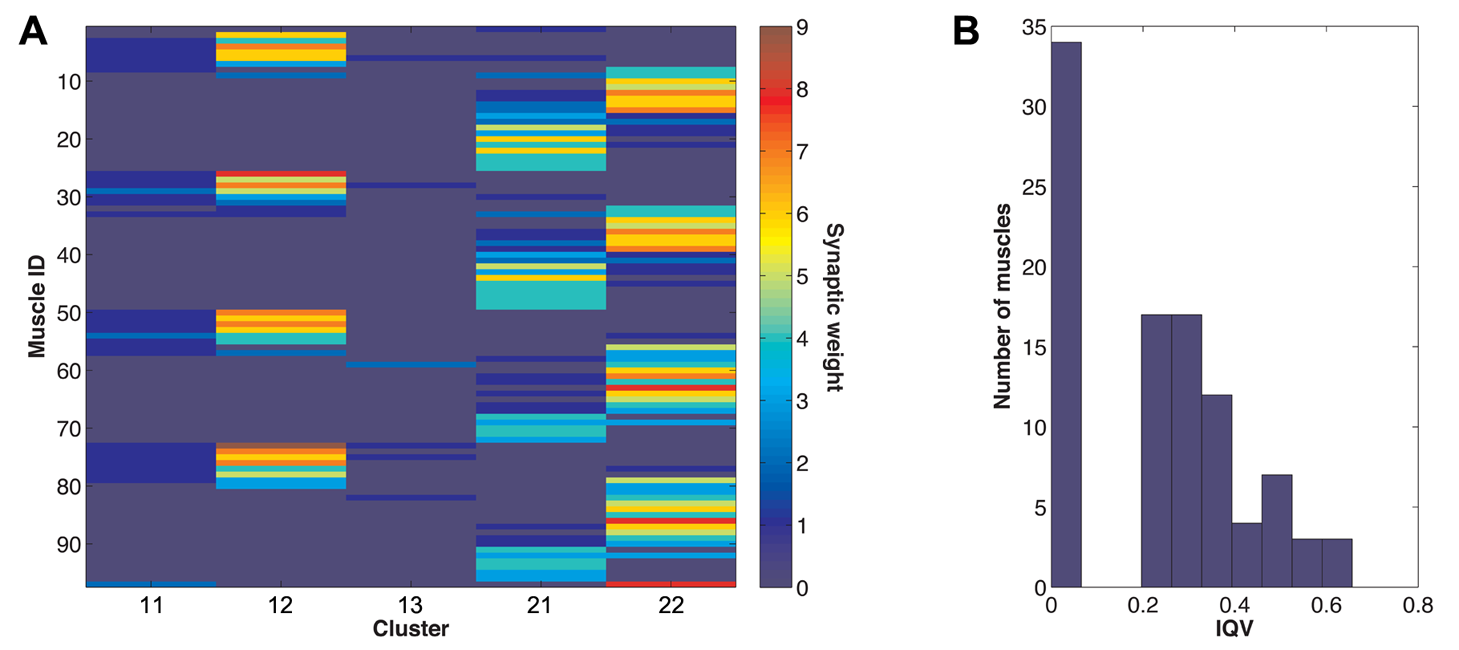

Supplement: Figure S5 — Association between muscles and the clusters. (A) Strength of attachment of each muscle to the clusters represented by synaptic weight linked to the clusters. (B) Distribution of diversity of linked clusters for each neuron measured using IQV. (0.15 MB TIF) [file pcbi.1001139.s005.tif]

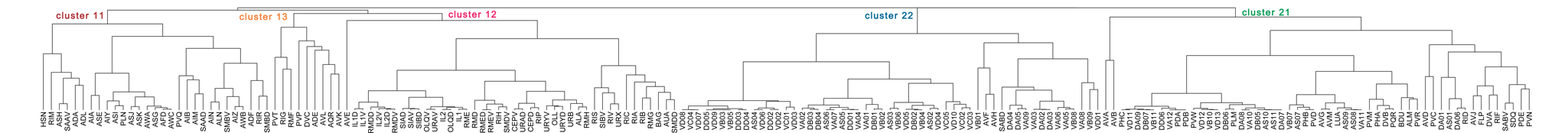

Supplement: Figure S6 — The complete community hierarchy of the 279 neurons. (0.12 MB TIF) [file pcbi.1001139.s006.tif]
