# Supplementary material for: Topological Cluster Analysis Reveals the Systemic Organization of the Caenorhabditis elegans Connectome
Source: PLoS Comput Biol. 2011 May 19;7(5):e1001139. doi: 10.1371/journal.pcbi.1001139 (PMC3098222; doi:10.1371/journal.pcbi.1001139)
Supplement: Text S1 — Supporting Information (0.07 MB DOC) [file pcbi.1001139.s011.doc]

**Supporting Information**

**Methods in detail**

**Spectral method for modularity-based community detection**

The complete neuronal wiring diagram of *C. elegans* through chemical synapses and electrical junctions (connectome) was considered to be a directed weighted network described by basic topological attributes including degree, weight, and strength [1]. A weighted asymmetric adjacency matrix was devised to illustrate the synaptic connections between 279 neurons. The matrix size was accordingly 279 × 279 and the sum of the weights of each element represented the number of synapses from one neuron to another.

To identify possible topological clusters in the *C. elegans* connectome, we used the modularity-based community detection algorithm proposed by Newman [2,3,4,5]. This algorithm assumes that the number and size of clusters are determined by the network itself and not by the experimenter. Moreover, modularity-based community detection methods may explicitly admit the possibility that no good division of the network exists, an outcome that is itself considered to be of interest for the light it sheds on the topology of the network.

The method was designed to obtain optimal partitioning of a network that maximizes the modularity *Q* over the possible divisions. The modularity *Q* was expressed in terms of the eigenvectors of a characteristic matrix for the network, i.e., the modularity matrix *B*, as follows:

# $Q=\frac{1}{4W}s^{T}(B+B^{T})s$, [1] $B_{ij}=A_{ij}-\frac{S_{i}^{in}S_{j}^{out}}{W}$, [2]

where *A* is the adjacency matrix of a directed, weighted network, *S_i_^in^* and *S_i_^out^* indicate incoming and outgoing strengths, respectively, of neuron *i* and $W=\sum_{ij} A_{ij}=\sum_{i} S_{i}^{in}=\sum_{i} S_{i}^{out}$ is the global sum of the weights of all dyads. Hence, *B_ij_* becomes a measure of the extent to which the number of connections from neuron *j* to neuron *i* are prominent in comparison with a randomized network. The modularity *Q* is proportional to the number of edges falling within clusters minus the expected number in an equivalent network with edges placed at random. The *Q* value can be either positive or negative, with positive values indicating the existence of cluster structure. Thus, true cluster structure can be found by looking for the divisions of a network that have positive, preferably maximal Q values.

The assignment of a neuron to a specific cluster is determined by setting each element of vector *s* as 1 or -1, each of which represents the neuron’s affiliation with a distinct cluster. We can maximize the *Q* value by choosing an appropriate division of the network or, equivalently, by choosing the proper value of the index vector *s*. It is possible to achieve this goal with the eigenvectors of the modularity matrix *B*, i.e., by making the maximum parallel *s* to the eigenvector with the largest eigenvalue [4,5]. The simplest way to do this is to assign 1 or -1 to *s_i_* using the same sign as the element of the eigenvector in the corresponding position. We computed the leading eigenvector of the modularity matrix *B* and divided the vertices into two clusters according to the signs of the elements in this vector.

After achieving the division of two grand clusters, repetition of the procedure on the previously derived clusters to find the optimal subdivisions with maximal *ΔQ* provides more than two clusters. Consequently, the method reveals the binary tree structure of the clusters in a network. The mathematical details for undirected network decomposition were presented by Newman et al. [4], and those of the generalized version for directed network decomposition were demonstrated by Leicht and Newman [5]. To avoid micro-sized clusters with extremely small membership, we aggregated the resulting clusters with less than 5 members with other clusters that shared the closest common ancestor at the cluster branching tree (e.g., cluster 111 and cluster 112 were aggregated as cluster 11).

**Fast unfolding algorithm of community detection**

The fast unfolding algorithm of community detection [6] was claimed to be one of the most accurate and fast community algorithms to date [7]. This algorithm follows the standard greedy procedure at the nodal level by accumulating individual nodes into larger-sized clusters in the direction of increasing modularity. After this procedure reaches a local optimum, the algorithm builds a new network having previously assigned communities as its nodes; links between new nodes are weighted links exchanged between the communities. This repeats the same procedure done at the nodal level in the second phase and stops the procedure as it reaches another local optimum.

**Obtaining a null network**

We used a weighted edge-rewiring algorithm [8] to obtain null networks for the original connection. At each time step, two randomly selected connections were exchanged, preserving both the out-strength of every node in the original weighted, directed network and the connectedness of the whole network. Each edge was rewired 10 times on average. We obtained 100 null networks and compared their modularity values with that of the original network.

**Variation of information for quantifying differences in community structure**

A measure called the variation of information was used to derive the extent of the difference between two cluster divisions of a network. Karrer, Levina, and Newman [9] transplanted a measure from information theory to assess the differences between two distinct node classifications. Variation of information between two node classifications was expressed as follows:

$V(C,C^{'})=V(X,Y)=H(X|Y)+H(Y|X)$, [3]

where *X* and *Y* denote the vectors representing the cluster assignment of community divisions *C* and *C’*, respectively, *H(X|Y)* is the conditional entropy indicating the amount of additional information needed to describe *C* given *C’*, and *H(Y|X)* indicates the opposite condition. Hence, larger variation of information between two cluster assignments, (i.e., more information is needed to describe one another) results in a larger difference between the cluster assignments. The value becomes zero when two cluster assignments are identical, whereas the maximal possible value between two node assignments for an N-node network is log N.

**Spatial density distributions for clusters along the anterior-posterior body axis**

Spatial density distributions for clusters were calculated along the anterior-posterior body axis based on the sum of the delta functions, which have an infinite value at the location of neurons and zero at other locations. The sum of the delta functions, represented the horizontal positions of neurons belonging to the same cluster, was smoothed by the kernel density function. The density value was normalized to the maximal value in each cluster and consequently ranged from 0 to 1.

**Index of qualitative variation (IQV)**

When a cluster consists of neurons with different types or affiliations, the degree of qualitative variation can be quantified as follows [10]:

# $IQV=\frac{n}{n-1}\left(1-\sum_{k=1}^{n} p_{k}^{2}\right)$, [4]

where *p_k_* is the proportion of neurons in the cluster with quality (neuronal type or ganglion type in this study) *k*, and *n* is the variability in the types of neurons. If a cluster is composed of few dominant types, IQV would be close to 0; in the opposite case, the value would reach 1. *(n/(n-1))* is a scaling factor.

**Functional cartography of members in a network**

To quantify the intra-cluster and inter-cluster connections of neurons in a modular network, we estimated the within-module weight (Z) and the participation coefficient (*P*) of the symmetrized weighted network [3]. The within-module weight (*Z*) measures how well-connected a neuron is to other neurons within the cluster with respect to the average of the neurons in that cluster. For a weighted symmetric network, *z* is described as:

# , [5]

where *w_i_* is the sum of weights of neuron *i* bridged with other neurons in its cluster *s_i_*, *w_si_* is the average of *w* over all of the neurons in *s_i_*, and σ_w_ is the standard deviation of *w* in *s_i_*.

The variability of connections of a neuron with neurons belonging to other clusters is quantified by the participation coefficient *P_i_*, which is, roughly speaking, an IQV for measuring the diversity of the partnership of the neuron. We defined the participation coefficient *P_i_* of a neuron *i* as:

# $P_{i}=1-\sum_{s=1}^{N_{M}} \left(\frac{w_{is}}{S_{i}}\right)^{2}$, [6]

where *w_is_* is the sum of weights of neuron *i* to neurons in module *s*, and *S_i_* is the strength of neuron *i*.

**Hub and authority scores of clusters**

To determine which cluster was responsible for sending information and which cluster received information from authoritative sources, we calculated the authority and hub scores for the network of clusters (an adjacency matrix representing the direction and synaptic weights between clusters). These parameters are modifications of eigenvector centrality and are very effective at determining central nodes in directed networks [11]. To derive the hub score of a cluster, a value that indicates the relative amount of outward synapses heading to clusters having high authority scores, we obtained the corresponding element in the normalized eigenvector of the matrix *AA^T^*, which has the largest eigenvalue. On the other hand, we extracted the normalized eigenvector of matrix *A^T^A* having the largest eigenvalue to derive the authority scores, which indicate the relative amount of inward synapses oriented from clusters having high hub scores. Therefore, a good hub cluster directs to many good authorities, and a good authority is directed to many good hubs.

**Additional Results**

**Robustness of optimality of the community assignment vector**

To examine the robustness of the optimality of the primarily-analyzed community assignment vector obtained by the simulated annealing method with external constraints, we compared the modularity scores achieved by applying the vector to slightly perturbed networks and the scores derived by aplying spectral methods to the perturbed networks. Assuming the presence of missing and incorrect information in the dataset, we selected *p*% of the overall connections and added Gaussian noise with a mean of 0 and a standard deviation *l* (unit: number of synapses). We showed that in the *p*:0~5% and *l*:0~2 regions, the two types of modularity values differed by only 0.011 (± 0.002), whereas the optimal modularity value for the unperturbed network was 0.4902.

**Neuronal types and neurotransmitters in clusters**

We determined whether specific clusters of neurons obtained from the modularity-based community detection algorithm had an intimate correlation with their neuronal types in terms of neurotransmitters. Four of 5 clusters did not display dominant neurotransmitter types (Figure S2). In cluster 22, which includes motor neurons as a dominant neuronal type, most members secreted acetylcholine or GABA.

**Association between clusters and cell lineage distance between neurons**

We examined whether an association existed between the cluster assignment and cell lineage distance between neurons. The lineage distance between two neurons was defined as the sum of distances from two neurons to their nearest common ancestor in the cell lineage tree of *C. elegans* [6]. Because neurons in C. elegans are divided into the left and right sides of the worm in the earliest stage of development, we calculated, for each of the 5 clusters, the average lineage distance of 93 left-side neurons that belonged to the same cluster. The average lineage distance of neurons belonging to the same cluster was 15.09 ± 2.73, whereas the global average of the 93 neurons was 15.26. Therefore, this result suggests that structural clusters whose memberships are determined by relative connection densities are not associated with neuronal phylogeny.

**Distribution of poly-synapses**

To assess the distribution of polyadic synaptic connections in the connectome, we drew a heat map for the ratio of poly-synaptic weights to overall chemical synaptic weights in each cluster pair (Figure S4). Values in the matrix represent the values minus the global ratio (0.55). We could not find an inter-cluster pair or an intra-cluster element that had a positive value for the ratio. One interesting finding was that cluster 22, which had motor neurons as a majority of its members, had a very low ratio of poly-dyadic synapses (-0.48) compared to the global mean. However, this effect washes out when we consider that 41% of NMJ connections, whose majority are polyadic connections, are bridged with the neurons in cluster 22.

**Topological association between the muscles and the clusters**

Since the connection profiles between muscles by gap junctions are not available at the moment (12 Liu et al). We could not include the muscles as additional nodes of the network used for the community detection analysis which was initially proposed to detect community structure of an one mode non-bipartite network. Instead we measured the topological proximities between each muscle and the clusters. Figure S5A presents the connection density of the 97 muscles to the neurons in each cluster. As quantitatively assessed in Figure S5B, the majority of the muscles have a single dominant cluster association. 35% of the muscles have NMJ connections with exactly one cluster and the rest of the muscles exhibit far less IQV values than 0.8 which is the value achieved when cluster association is randomly assigned. The clusters with which subsets of muscles exhibit single dominant associations are the 12, 21 and 22 clusters. Unlike the other two clusters, these clusters have motor neurons as major members. The positions of the muscles, not their functional identities, were the determining factors of which clusters of neurons that they are associated with. Anterior muscles were associated with the neurons belonging to the 12 cluster; the muscles in the mid body with those in the 21 cluster, and the posterior muscles with those in the 22 cluster. This anterior-to-posterior association is consistent with the information flow derived from our cluster analysis of neurons without taking NMJs into consideration.

**References**

1. Barrat A, Barthelemy M, Pastor-Satorras R, Vespignani A (2004) The architecture of complex weighted networks. Proc Natl Acad Sci U S A 101: 3747-3752.

2. Clauset A NM, & Moore C (2004) Finding community structure in very large networks. Phys Rev E 70: 066111-066113.

3. Guimera R, Amaral LAN (2005) Cartography of complex networks: modules and universal roles. J Stat Mech 2001. P02001.

4. Newman MEJ (2006) Finding community structure in networks using the eigenvectors of matrices. Phys Rev E 74: 36104.

5. Leicht EA, Newman ME (2008) Community structure in directed networks. Phys Rev Lett 100: 118703.

6. Blondel VD, Guillaume JL, Lambiotte R, Lefebvre E (2008) Fast unfolding of communities in large networks. J Stat Mech P10008.

7. Lancichinetti A, Fortunato S (2009) Community detection algorithms: a comparative analysis. Phys Rev E 80(5 Pt 2):056117.

8. Maslov S, Sneppen K (2002) Specificity and stability in topology of protein networks. Science 296(5569):910-913.

9. Karrer B, Levina E, Newman MEJ (2008) Robustness of community structure in networks. Phys Rev E 77: 046111-046119.

10. Lichtman JW, Livet J, Sanes JR, Contact NPG (2008) A technicolour approach to the connectome. Nat Rev Neurosci 9(6):417-422.

11. Kleinberg JM (1999) Authoritative sources in a hyperlinked environment. J Assoc Comput Mach 46(5):604–632.
